# Confocal imaging of mouse mandibular condyle cartilage

**DOI:** 10.1038/srep43848

**Published:** 2017-03-07

**Authors:** Y. He, M. Zhang, A. Y. Huang, Y. Cui, D. Bai, M. L. Warman

**Affiliations:** 1 Orthopaedic Research Laboratories, Department of Orthopaedic Surgery, Boston Children’s Hospital and Department of Genetics, Harvard Medical School, Boston, MA, USA; 2 State Key Laboratory of Oral Diseases, Department of Orthodontics, West China Hospital of Stomatology, Sichuan University, Chengdu, China; 3 Howard Hughes Medical Institute, Boston Children’s Hospital, Boston, MA, USA.

## Abstract

Mice are commonly used to study the temporomandibular joint (TMJ) and to model human TMJ disease. However, evaluating TMJ pathology in mice using standard histologic methods is time consuming, labor intensive, and dependent upon investigators’ expertise at consistently orienting and sectioning across tiny specimens. We describe a method that uses confocal microscopy to rapidly and reliably assess indicators of mandibular condyle cartilage pathology in mice. We demonstrate the utility of this method for detecting abnormalities in chondrocyte distribution in mice lacking lubricin (*Prg4*), the major boundary lubricant of articular cartilage. We further show that the method can provide information about recombination sites and efficiency in mandibular cartilage for Cre-driver strains. Because specimen preparation and data acquisition with confocal microscopy are simple and fast, the method can serve as a primary screening tool for TMJ pathology, before proceeding to complicated, time consuming, secondary analyses.

The temporomandibular joint (TMJ) is a specialized synovium-enclosed articulating structure composed of the mandibular condyle, an interposed fibrocartilaginous disc, and the glenoid fossa^1^. The TMJ is needed for eating and speaking, making it among the most used joints. Like other synovial joints, the TMJ can be affected by inflammatory, traumatic, infectious, developmental, and neoplastic diseases. In fact, at least one sign of TMJ disease is present in 40 to 75% of US adults^2^.

Mice and humans share many developmental and molecular pathways, which led to mice being used for modeling human TMJ diseases^3^. However, methods for evaluating the consequences of TMJ disease in mice are challenging because of the small size of this joint. Therefore, to facilitate TMJ studies in mice, we developed a simple and fast confocal microscopic imaging method for the mandibular condyle. Herein, we describe the imaging method and demonstrate its utility by studying wild-type (WT) and lubricin (*Prg4*) deficient mice, the latter modeling the human genetic disease Camptodactyly-Arthropathy-Coxa vara- Pericarditis (CACP) syndrome^4^, and by determining the pattern of Cre-mediated recombination for the *Acan*^CreERt2^ mouse strain^5^. This strain, which has a tamoxifen-inducible Cre-recombinase knocked into the Aggrecan (*Acan*) locus, is commonly used to study articular cartilage.

## Results

### Confocal imaging of a mouse mandibular condyle

**Figure 1:**
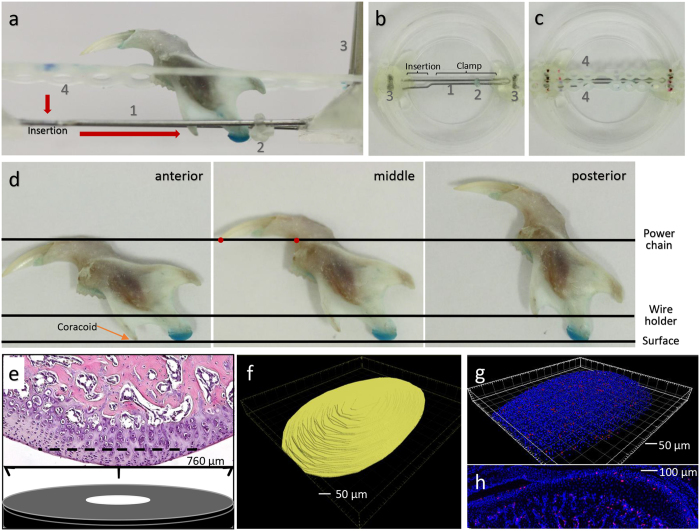
Confocal imaging of the mouse mandibular condyle. (**a**) Side view of the condyle specimen holder showing [1] a 0.05 mm diameter stainless steel wire (Ormco, Orange, CA) that has been bent 180° in order to clasp the neck of the condyle and keep it in line with the coracoid process, [2] a plastic stop to keep the condyle from moving, [3] pairs of 0.018 × 0.025 inch rectangular arch wires (Orthopli Corp, Philadelphia, PA) implanted vertically in dental resin to grasp [4] two parallel J120 closed clear power chains (Rocky Mountain Orthodontics, Denver, CO) which keep the ramus in line with the condyle. (**b**) Top view of the specimen holder demonstrating how the wire has been bent to efficiently insert and clasp the condylar neck. (**c**) Top view of the specimen holder with the 2 power chains added. (**d**) Side views indicating how the condyle (stained blue with toluidine) and coracoid processes are oriented to image the condyle’s anterior, middle, and posterior regions. (**e**) H&E stained sagittal section containing the middle region of the condyle with length and depth of the confocal imaging field obtained by confocal objective indicated. (**f**) Bird’s eye view of a 3D reconstruction depicting the confocal imaged volume (yellow) of the middle condylar region from a 6-week-old male mouse. (**g**) Bird’s eye view of a 4-week-old mouse condyle showing centroids of DAPI stained (blue) and EdU containing (pink) chondrocyte nuclei. (**h**) Sagittal section through the contralateral mouse condyle imaged for DAPI (blue) and EdU (pink) fluorescence, showing that the frequency and distribution of EdU-containing chondrocytes are comparable to that found with confocal imaging.

Each specimen could be loaded and imaged for two fluoroscopic channels in less than 6 minutes. Representative three dimensional (3D) reconstructed confocal images for the middle region of the mandibular cartilage from a 6-week-old WT mouse are shown in Fig. 1. DAPI stained and EdU containing chondrocyte nuclei were reliably detected to a depth of 50 μm beneath the articulating surface. Therefore, measurements of chondrocyte number and condyle volume were performed using a 760 × 760 × 50 μm field. In order to determine the coefficient of variation (CV) for counting the number of chondrocyte nuclei, for measuring cartilage volumes, and for determining the number of cells per unit volume, we imaged condyles from 3 WT mice 10 times each. Every CV was less than 0.11 and most of them were less than 0.05, which led us to conclude that imaging a specimen 3 times would be sufficient to obtain an average value that would fall close to the true value. We then imaged 10 WT 6-week-old male mice. In these genetically identical mice, the standard deviations (SD) for cell number and cartilage volume were ∼14% of the mean, whereas the SDs for the number of cell nuclei/cartilage volume (hereafter termed “cell density”) were ∼7% of the mean for the cartilage volume lying within 10 μm of the articular surface, the “superficial cell density” (Table 1 and Supplementary Table 1). We chose the volume 10 μm closest to the condylar surface based on prior work that showed these cells express *Dkk3* whereas deeper cells do not^6^,^7^. Thus, as few as 4 mice per group could be used to detect a ≥ 20% difference in superficial cell density between experimental and control animals (Table 1). All subsequent experiments considered measures of cell density, since larger sample sizes would be required to observe significant differences in chondrocyte number or cartilage volume (Supplementary Table 1). The other cell density measure we considered was the number of nuclei in the imaged cartilage volume from 10 μm below the surface to the bottom of the imaged cartilage, the “deeper cell density”.

The Bland-Altman analyses revealed no significant biases in any of the measures used to calculate either the superficial or deep cell densities (Supplementary Figure 2 and Table 2).

### Confocal microscopy confirms chondrocyte loss in mice lacking lubricin

**Figure 2:**
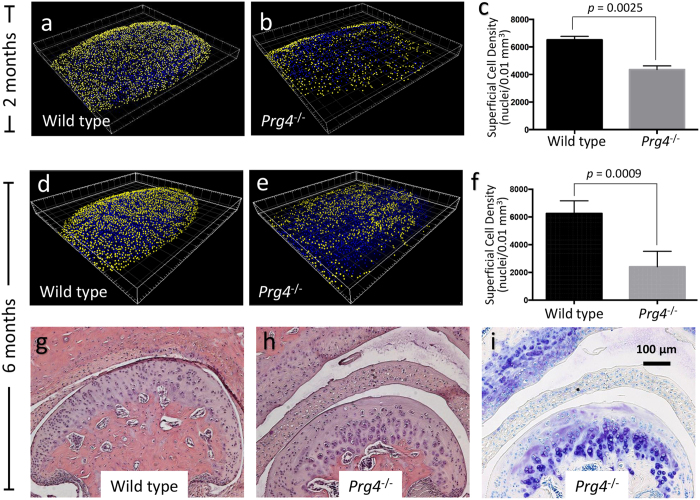
Confocal imaging detects differences in condylar shape and chondrocyte density between wild-type and *Prg4* knockout mice. (**a**) 3D reconstruction depicting nuclear centroids in the middle region of a 2-month-old wild-type mouse (superficial cell nuclei, that is those within 10 μm of the condylar surface, are pseudocolored yellow and deeper cell nuclei are pseudocolored blue). (**b**) 3D reconstruction of the middle region of a 2-month-old *Prg4* knockout (*Prg4*^−/−^) mouse with the same pseudocoloring as in panel A. Note there are fewer surface chondrocytes in the knockout mouse condyle. (**c**) Bar graph showing the mean (+SD) superficial cell densities for the middle region of 2-month-old Wild-type (n = 3) and *Prg4*^−/−^ (n = 3) mice. (**d** and **e**) Similar 3D reconstructions as in panels A and B, but in 6-month-old Wild-type and *Prg4*^−/−^ mice, respectively; note the condyle is now flattened and widened in the knockout mouse. (**f**) Bar graph showing the mean (+SD) superficial cell densities for the middle region of 6-month-old Wild-type (n = 4) and *Prg4*^−/−^ (n = 5) mice. (**g**) H&E stained coronal section through the middle region of the TMJ from a 6-month-old wild-type mouse. (**h**) H&E stained coronal section through the middle region of the TMJ from a 6-month-old *Prg4*^−/−^ mouse. Note the knockout mouse has a thicker fibrocartilaginous disc and less superficial chondrocyte nuclei compared to the wild-type mouse. (**i**) Toluidine blue stained TMJ section from the knockout mouse confirming that the cartilage surface has become acellular.

It has previously been reported, using standard histology, that lubricin knockout mice develop age-related abnormalities in their TMJs, including widening and flattening of condylar surface with superficial chondrocyte loss^1^,^8^,^9^. Our confocal imaging detected these same changes in *Prg4* knockout mice without requiring decalcification, paraffin embedding, sectioning, and staining (Fig. 2).

### Confocal microscopy reveals non-uniform Cre-expression in the TMJ of *Acan* ^CreERt2^ mice

**Figure 3:**
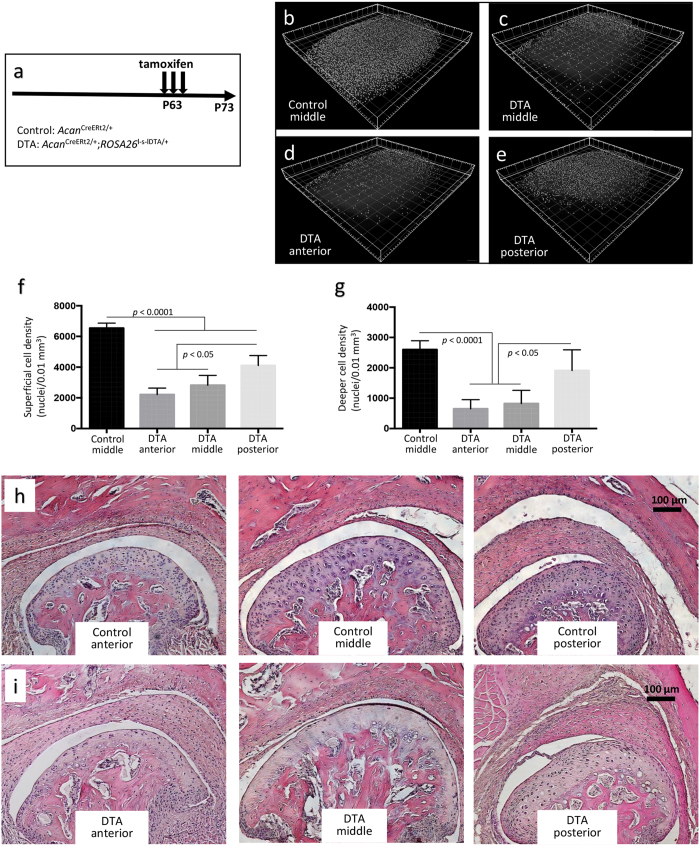
Confocal imaging suggests a difference in *Acan*^CreERt2^ mediated chondrocyte killing from the anterior to posterior region of the TMJ. (**a**) Schematic depicting the experimental timeline for inducing diphtheria toxin (DTA) expression with a tamoxifen-inducible Cre knocked into the *Acan* locus. Mice were given tamoxifen for 3 consecutive days at P63 (9-weeks-old) and studied at P73. (**b**) 3D reconstruction depicting nuclear centroids in the middle region of a 10-week-old control (*Acan*^CreERt2/+^) mouse, pseudocolored white. (**c**) 3D reconstruction of the middle region from a 10-week-old DTA (*Acan*^CreERt2/+^; *ROSA26*^*l*-s-lDTA/+^) mouse; i.e., a mouse induced to express diphtheria toxin when 9-weeks-old. (**d** and **e**) 3D reconstructed images from the anterior (**d**) and posterior (**e**) regions of the same DTA mouse. Note there are fewer nuclei in the DTA condyle compared to the control condyle, and fewer nuclei in the anterior and middle regions compared to the posterior region of the DTA condyle. (**f**) Bar graph showing the mean (+SD) superficial cell densities for the middle region of control mice (n = 6) and the anterior, middle, and posterior regions of DTA mice (n = 4). Note, in control mice the surface cell density is the same for the anterior, middle, and posterior regions (data not shown). In the DTA mice, there is significantly less cell loss in the posterior compared to anterior and middle regions. (**g**) Bar graph showing the mean (+SD) deeper cell densities for the middle region of control mice (n = 6) and the anterior, middle, and posterior regions of DTA mice (n = 4). Note, deeper cell loss is greater in the anterior and middle regions of the DTA mouse condyle compared to the posterior region. (**h** and **i**) H&E stained coronal sections through the anterior, middle, and posterior regions of the TMJs from a control and a DTA mouse, respectively. Note the posterior region of the DTA mouse has less cell loss than the anterior and middle region. Also, note there is more killing of deeper cells compared to superficial cells in anterior and middle regions of the DTA mouse condyle.

**Figure 4:**
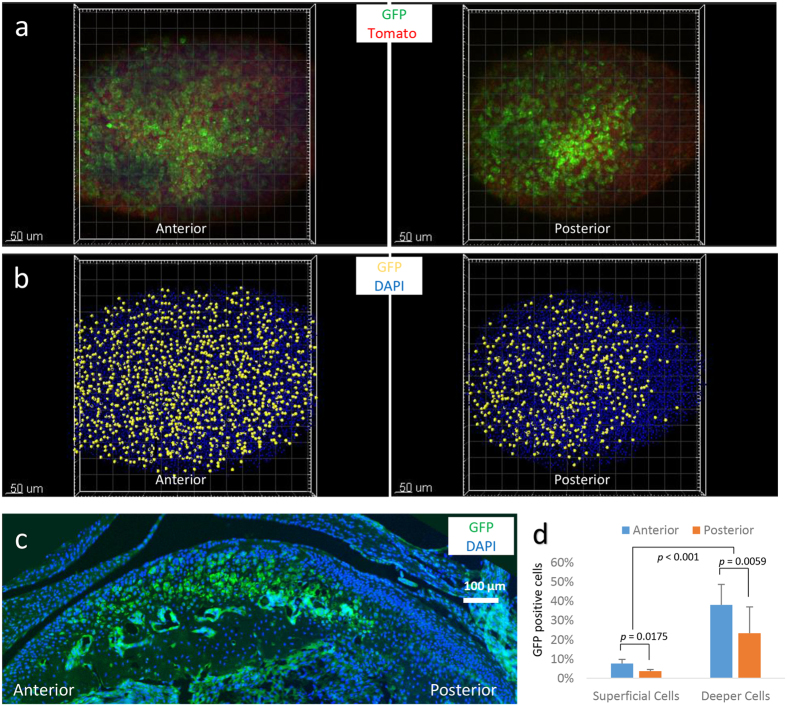
Confocal imaging confirms a difference in *Acan*^CreERt2^ mediated recombination from the anterior to posterior region of the TMJ. (**a**) Flattened confocal images indicating green fluorescent protein (GFP) expressing chondrocytes in the anterior and posterior regions of a *Acan*^CreERt2/+^; *ROSA26*^mTmG/+^ mouse that was induced to express membrane bound GFP instead of tomato fluorescent protein when 9 weeks-old. Twenty-five images taken at 2 μm intervals to a depth of 50 μm were flattened for this image. (**b**) Flattened confocal images representing centroids of GFP expressing chondrocytes that have been pseudocolored yellow in the anterior and posterior regions of the same mouse depicted in panel A; non-GFP expressing chondrocyte nuclei are pseudocolored blue. Note the uniform distribution of nuclei from GFP expressing chondrocytes in the anterior region contrasts with the asymmetric distribution observed in the posterior region. (**c**) Fluorescence microscopic image of a sagittal section from a similarly treated mouse confirming there are more GFP expressing chondrocytes in the anterior and middle regions of the condyle compared to the posterior region; DAPI-stained nuclei are pseudocolored blue. (**d**) Bar graph showing the mean (+SD) percentages of GFP expressing superficial cells and deeper cells in the anterior and posterior regions of the mandibular condyle from tamoxifen-treated *Acan*^CreERt2/+^; *ROSA26*^mTmG/+^ mice (n = 4). Note the fraction of GFP expressing cells is greater in the anterior compared to posterior region and greater among deeper cells compared to superficial cells.

The *Acan*^CreERt2^ mouse strain is frequently used to conditionally recombine genes with floxed alleles in articular cartilage, including the TMJ^10^. We observed that *Acan*^CreERt2^ is not uniformly active throughout the mandibular cartilage. We suspected this after we used the *Acan*^CreERt2^ strain to induce diphtheria toxin (DTA) expression in order to kill TMJ chondrocytes. We administered 3 daily doses of tamoxifen to P63 (9-week-old) *Acan*^CreERt2/+^; *ROSA26*^l-s-lDTA/+^ mice and then evaluated their mandibular condyles at P73 (Fig. 3). In control mice, we observed no difference in chondrocyte density between the anterior, middle, and posterior condylar regions (data not shown). However in the *Acan*^CreERt2/+^; *ROSA26*^l-s-lDTA/+^ tamoxifen-treated mice, significantly more chondrocyte loss occurred in anterior and middle regions compared to the posterior region (Fig. 3). We repeated the experiment using mice with a floxed fluorescent reporter allele (*ROSA26*^mTmG^) and again found more *Acan*^CreERt2^ activity in the anterior and middle regions compared to the posterior region and in deeper chondrocytes compared to superficial chondrocytes (Fig. 4). These confocal microscopic observations were confirmed when we examined the condyles with standard histologic methods (Figs 3 and 4).

## Discussion

The mouse is commonly used to model human TMJ disease^3^. Mice can also be used to identify diseases that cause TMJ pathology, which may be asymptomatic in patients. For example, lubricin deficiency causes striking and profound TMJ pathology in mice^1^,^8^,^9^, and in fish^11^, but the clinical literature does not mention TMJ involvement in patients. Even in mice, TMJ pathology may be missed because most scientists lack expertise in evaluating the mouse TMJ. Therefore, to facilitate studies in the mouse, we adapted a confocal imaging technique for studying long bone articular cartilage^12^. Because the mouse mandibular condyle is small, we designed a simple positioning apparatus to image the condyle’s anterior, middle, and posterior regions (Fig. 1 and Supplementary Figure 1). We showed that specimen processing requires minimal sample handling and that the condyle can be imaged to a depth of 50 μm quickly and reproducibly (Fig. 1, Table 1, and Supplementary Table 1).

Using wild-type mice, we determined that cell density was similar across the anterior, middle, and posterior condylar regions (data not shown), and higher nearer the surface (Supplementary Figure 1). We then compared WT and lubricin (*Prg4*) knockout mice to show that few mice were needed to detect a significant difference in cell density (Fig. 2). Importantly, we confirmed our confocal imaging results using histologic methods, and showed the latter agreed with results obtained by other investigators^1^,^8^,^9^. Taken together, our findings suggest that confocal microscopy can serve as a rapid screening tool for TMJ disease in mice. Mice found to have condylar abnormalities by confocal microscopy can then have their entire TMJ examined in detail using more extensive, but more costly, histologic methods.

Another use of condylar confocal microscopy will be to determine patterns of Cre-recombinase activity for driver strains. We demonstrated this using the *Acan*^CreERt2^ allele, which is used to conditionally alter genes in cartilage. When we administered tamoxifen to 9-week-old mice with an *Acan*^CreERt2^ allele and a reporter allele (i.e., DTA or mTmG), we observed significantly more Cre-recombinase activity in the anterior compared to the posterior region of the condyle (Figs 3 and 4). We also observed more Cre-recombinase activity in deeper compared to superficial chondrocytes. These confocal data agree with data for *Acan*^CreERt2^ that other investigators obtained using standard histology and LacZ staining^10^, and are consistent with there being a greater abundance of fibrochondrocytes, compared to hyaline chondrocytes, near the surface and in and posterior region of the condyle. The confocal method extends these earlier findings by quantifying the rate of Cre-recombination (Figs 3 and 4) and by demonstrating that recombination efficiency varies depending upon the reporter allele (Figs 3 and 4). Specifically, we observed *Acan*^CreERt2^ was more efficient at recombining the *ROSA26*^l-s-lDTA^ allele than the *ROSA26*^mTmG^ allele. The latter allele appears more difficult to Cre-recombine than other *ROSA26* alleles^13^, perhaps because it contains two consecutive highly homologous cDNAs encoding fluorescent reporter proteins.

We are not the first to apply confocal imaging to the mouse mandibular condyle. For example, confocal microscopy has been used to evaluate sites of fluorescent protein expression in transgenic mice^7^. However, these investigators did not describe standardized methods for orienting their specimens or for collecting quantitative data. We used common dental supplies to build a specimen holder and developed a standard positioning system that enabled us to image the condylar surface quickly and at high resolution. Although there are other 3D imaging strategies for examining mouse cartilage *in situ*, including μMRI and μCT^14^, the advantages of confocal microscopy are its higher resolution compared to μMRI and its shorter image acquisition time compared to μMRI and μCT.

A limitation of our imaging method is that it does not examine the fibrocartilaginous disc or the glenoid fossa. Therefore, it will be less sensitive for screening mice whose disorders affect the disc and fossa before they affect the condyle. At present, we do not know when condylar involvement occurs in mouse models of TMJ. In the case of lubricin deficiency, condylar involvement occurs early even though the major sites of *Prg4* expression are in the disc and glenoid fossa^1^. Similarly, when TMJ partial discectomy is performed to induce disease, condylar involvement appears within weeks of the primary injury^15^. Therefore, we anticipate that our method will be useful for many mouse models in which TMJ disease develops and for other studies of TMJ development, such as the transdifferentiation of condylar chondrocytes into osteoblasts^16^. Other image quantification strategies^17^ may also improve the utility of our method. We look forward to other investigators extending our method by adding new fluorescent agents, image analysis algorithms, and mouse models of TMJ disease.

## Methods

All studies were performed in an AAALAC accredited facility in accordance with National Institutes of Health and Boston Children’s Hospital guidelines for research involving mice. The Institutional Animal Care and Use Committee at the Boston Children’s Hospital approved these studies.

### Experimental animals

WT and *ROSA26*^l-s-IDTA^ mice on a C57BL/6 J background, *ROSA26*^mTmG^ mice on a 129 × 1/SvJ background, and *Acan*^CreERt2^ mice were obtained from the Jackson Laboratories (Bar Harbor, ME), stock numbers 00664, 009669, and 007576, respectively. *Prg4* loss-of-function alleles, *Prg4*^−^ and *Prg4*^GT^, have been previously described^4^,^9^; these mice are also available from the Jackson Laboratories (stock numbers 025737 and 025740, respectively).

Cre-recombinase activity was induced in 9-week-old mice with the *Acan*^CreERt2^ allele by administering 3 daily intraperitoneal (IP) injections of tamoxifen (150 mg/kg/dose) diluted in corn oil (both from Sigma-Aldrich, St. Louis, MO).

Labeling of dividing cells was performed by administering a single 50 mg/kg dose of 5-ethynyl-2′-deoxyuridine (EdU) IP (Santa Cruz Biotechnology, Dallas, TX).

Experimental animals were killed by CO_2_ inhalation immediately prior to specimen recovery and processing.

### Specimen processing

After sacrifice, the skull and mandible were hemisected. To recover individual mandibular condyles, the overlying muscle was removed, the TMJ capsule opened, and the mandibular condyle was disarticulated from its glenoid fossa and fibrocartilaginous disc. The mandible was then fixed in 4% paraformaldehyde (PFA, Affymetrix, Cleveland, OH) at 4 °C. The contralateral mandible was either disarticulated and fixed or fixed *in situ* with the other components of the TMJ for whole mount sectioning, depending on the experiment.

### Histology

The right PFA-fixed hemi-mandible was decalcified with 14% EDTA (pH 7.5) for up to 14 days, depending upon the animal’s age. Samples were then paraffin embedded so that the condyle could be sectioned in the sagittal or coronal plane. Six μm thick sections were obtained and stained with hematoxylin and eosin (H&E) or toluidine blue using standard methods. Sections from equivalent regions of the TMJ were compared between animals.

For experiments assessing the efficiency of EdU labeling in WT mice and of Cre-mediated recombination in *Acan*^CreERt2/+^; *ROSA26*^mTmG/+^ mice, after the mandible was decalcified in EDTA, it was equilibrated in 15% followed by 30% sucrose before being embedded in O.C.T. compound (Sakura, Torrance, CA). Eight μm thick frozen sections from WT and *Acan*^CreERt2/+^; *ROSA26*^mTmG/+^ mice were then obtained in the sagittal plane. For EdU containing sections, the Alexa Fluor 647 Click-iT EdU imaging kit (Invitrogen, Carlsbad, CA) was using following the manufacturer’s directions. All frozen sections were stained by ProLong Gold antifade reagent with DAPI (4′,6-diamidino-2-phenylindole) (Molecular Probes, Eugene, OR) and imaged using a Nikon Eclipse 80i (Nikon, Sendai, Japan) fluorescence microscope with a CoolSNAP HQ2 camera (Photometrics, Tucson, AZ).

### Confocal microscopy

The non-decalcified left PFA-fixed hemi-mandible was placed in PBS with DAPI (Thermo-Fisher Scientific, Eugene, OR, 10 μg/ml) at 4 °C for at least 24 hours. For imaging cells that had incorporated EdU during cell division, the fluorophore Alexa 647 (Thermo-Fisher Scientific, Eugene, OR) was covalently linked to EdU-containing DNA as previously described^12^.

We fabricated a simple specimen holder using commonly available dental equipment (Fig. 1a–c). Condyles were loaded in the specimen holder, submerged in PBS, and imaged at 2 μm intervals to the depth of 50 μm. Three different orientations were tested in order to compare data for the anterior, middle, and posterior regions (Fig. 1d–f). Images were obtained with a Zeiss LSM780 confocal laser scanning (CLS) microscopy system (Carl Zeiss, Germany) with 20x Plan Apochromat NA 0.8 WD 550 mm dry objective, and a scanning speed of 6 as described previously^12^. When counting chondrocytes, we first determined the depth at which DAPI stained nuclei were clearly visible. When then determined the cartilage volume that extended to this depth. IMARIS software (Bitplane, Switzerland) was then used to generate 3D image reconstructions, calculate chondrocyte number and cartilage volumes, and determine the number of cell nuclei in volumes at different depths beneath the surface as previously described^12^.

### Statistical Analyses

To assess the agreement and correlation of our confocal imaging method, the left and right condyles of 10 mice were measured and analyzed by the Bland-Altman method. The two-sided t-test was used to supplement the agreement between the measurement of left and right condyles. The minimal sample sizes to detect significant differences under the two-sided t-test were estimated assuming equal numbers of mice in case and control groups. Student’s t-test and a linear regression model were applied for comparisons between experimental and control groups. At least 3 animals per group were studied, and *p*-values < 0.05 were considered statistically significant.

## Additional Information

**How to cite this article:** He, Y. *et al*. Confocal imaging of mouse mandibular condyle cartilage. *Sci. Rep.* **7**, 43848; doi: 10.1038/srep43848 (2017).

**Publisher's note:** Springer Nature remains neutral with regard to jurisdictional claims in published maps and institutional affiliations.

## Supplementary Material

Supplementary Tables and Figures

## Figures and Tables

**Table 1:** Calculated sample sizes needed to detect 10% to 50% differences in superficial cell density and deeper cell density in the mandibular cartilage of 6-week-old male C57Bl/6J mice.

Measure (nuclei/0.01 mm^3^)	Mean	Standard Deviation	Percent Change	Effect Size (change/SD)	Sample size requirements	
					80%	90%
Superficial Cell Density	6,456	437	10	1.48	8	11
			20	2.95	3	4
			30	4.43	2	3
			40	5.91	2	2
			50	7.39	2	2
Deeper Cell Density	3,245	384	10	0.85	23	30
			20	1.69	7	8
			30	2.54	4	4
			40	3.38	3	3
			50	4.23	2	3

The control mean and SD were obtained from 3 independent measurements on 10 pairs of mandibular condyles from C57Bl/6J mice. Note only 4 mice provide 90% power for detecting a 20% difference in superficial cell density, and 8 mice are required to detect a similar difference in deep cell density.
