# Supplementary material for: Confocal imaging of mouse mandibular condyle cartilage
Source: Sci Rep. 2017 Mar 7;7:43848. doi: 10.1038/srep43848 (PMC5339703; doi:10.1038/srep43848)
Supplement: Supplementary Tables and Figures [file srep43848-s1.pdf]

Confocal imaging of mouse mandibular condyle cartilage

Y. He[1,2,+], M. Zhang[1], A.Y. Huang[1], Y. Cui[1], D. Bai[2], and M.L. Warman[1,3]*

Supplementary Tables and Figures

Supplementary Table 1

Variable	Control mean	SD	Percent change	Effect size (Δ/SD)	Sample size	
					80%	90%
Superficial Cell Number	24.03×10^2	3.33×10^2	10%	0.72	31	41
			20%	1.44	9	11
			30%	2.17	5	6
			40%	2.89	3	4
			50%	3.61	3	3
Deeper Cell Number	26.79×10^2	4.44×10^2	10%	0.60	44	59
			20%	1.21	12	15
			30%	1.81	6	8
			40%	2.41	4	5
			50%	3.02	3	4
Superficial Cartilage Volume (μm^3)	37.37×10^5	5.54×10^5	10%	0.68	35	47
			20%	1.35	10	13
			30%	2.02	5	6
			40%	2.7	3	4
			50%	3.37	3	3
Deeper Cartilage Volume (μm^3)	83.44×10^5	14.96×10^5	10%	0.56	51	69
			20%	1.12	14	18
			30%	1.67	7	9
			40%	2.23	4	5
			50%	2.79	3	4

Supplementary Table 1. Sample size requirements for cell numbers and cartilage volumes

Calculated sample sizes needed to detect 10% to 50% differences for superficial or deeper cell number, and for superficial or deeper cartilage volume, in the middle region of mandibular cartilage from 6-week-old male wild-type mice. The control mean and SD were obtained from 3 independent measurements on 10 pairs of mandibular condyles from C57Bl/6J mice. Sample sizes are powered to provide an 80 or 90% chance of detecting a significant difference between experimental and control mice.

Supplementary Table 2. Bland-Altman analysis of mandibular condyle

	Bias±SD	Limits of Agreement	P value
Superficial Cell Number	$(0.20 \pm 3.83) \times 10^2$	-7.46×10^2 to 7.85×10^2	0.82
Deeper Cell Number	$(-1.62 \pm 5.93) \times 10^2$	-1.35×10^3 to 1.02×10^3	0.36
Superficial Cartilage Volume (μm^3)	$(-0.67 \pm 61.70) \times 10^4$	-1.23×10^6 to 1.23×10^6	0.99
Deeper Cartilage Volume (μm^3)	$(8.37 \pm 171.37) \times 10^4$	-3.34×10^6 to 3.51×10^6	0.86
Superficial Cell Density (nuclei/μm^3)	$(7.47 \pm 51.92) \times 10^{-5}$	-9.64×10^{-5} to 11.13×10^{-5}	0.65
Deeper Cell Density (nuclei/μm^3)	$(-2.19 \pm 3.71) \times 10^{-5}$	-9.60×10^{-5} to 5.23×10^{-5}	0.08

SD=Standard deviation; one-sided t-test was used to calculate the p value between the left and right side

Supplementary Table 2. Bland-Altman analyses for cell number, cell volume, and cell density in the middle region of the condyle.

Statistics were based on data obtained from 3 independent measurements on 10 pairs of mandibular condyles from 6-week-old male C57Bl/6J mice.

Supplemental Figure1

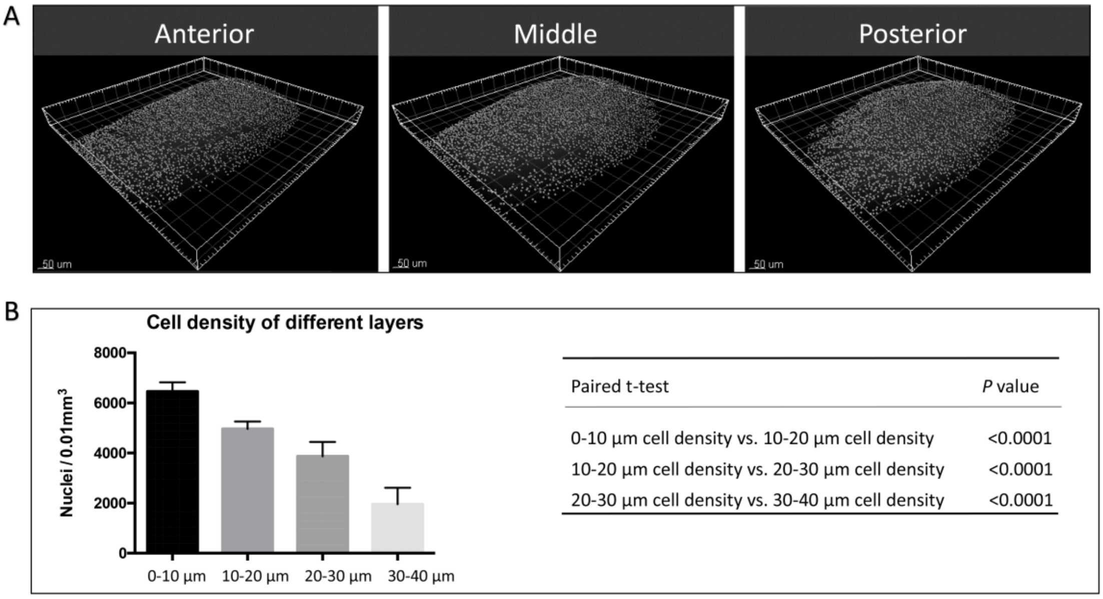

Supplementary Figure 1. Typical confocal images for the anterior, middle, and posterior regions and cell density determination for different depths beneath the cartilage surface.

(A) Bird's eye view of 3D reconstructed confocal images from the anterior, middle, and posterior regions the mandibular condyle of a 6-week-old male mouse. Centroids of cell nuclei are pseudocolored white. Note the similarity in cell density for all regions.

(B) Bar graph depicting the mean (+ SD) cell densities for the middle condyle region

at different depths beneath the cartilage surface based on studies of 20 condyles (right and left) from 10 6-week-old male mice. Note the mean cell density decreases as the depth from the surface increases; also note the SD increases as the depth from the surface increases.

Supplemental Figure 2

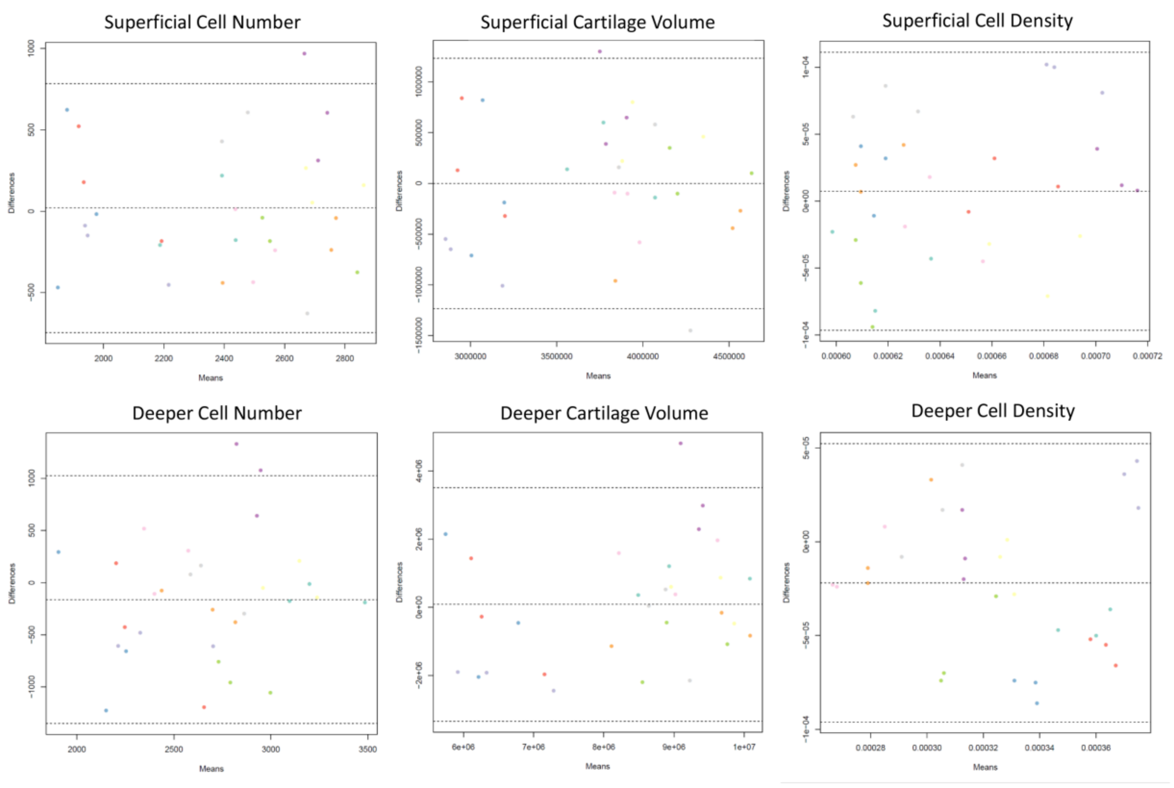

Supplementary Figure 2. Bland-Altman plots for cell number, cartilage volume, and cell density of mandibular condyle cartilage.

The six plots depict the data for cell number, cartilage volume, and cell density for the superficial and deeper regions obtained by imaging the middle region of the right and left condyles from 10 6-week-old male wild-type mice 3 times each. The different colors indicate different mice. The dotted lines indicate the mean value (± 1.96 SD).
